# Can Coexisting Allergic Rhinitis in Patients with Severe Eosinophilic Asthma Be a Prognostic Factor for Efficacy of Biological Therapy? Analysis of Eosinophilic Involvement

**DOI:** 10.3390/jcm15020587

**Published:** 2026-01-11

**Authors:** Edyta Jura-Szołtys, Joanna Glück, Ludger Klimek, Radosław Gawlik

**Affiliations:** 1 Clinical Department of Internal Diseases, Allergology and Clinical Immunology, Medical University of Silesia, 40-752 Katowice, Poland; jgluck@sum.edu.pl (J.G.);; 2 Center for Rhinology and Allergology, 66205 Wiesbaden, Germany

**Keywords:** eosinophil, allergic persistent rhinitis, non-allergic persistent rhinitis, bronchial asthma, biological treatment

## Abstract

Chronic rhinitis is induced by endotype-diverse inflammatory processes, which complicates effective therapeutic management. According to the current principles of personalized medicine, which also apply to the management of rhinological disorders, the best therapeutic results can be achieved after targeted treatment preceded by analysis of the patient’s endotype. Analysis of immune and cellular mechanisms allows for the use of biological treatment, and its effects provide new information on inflammatory processes in the nasal mucosa. The effects of biological treatment may be particularly interesting in the case of mixed endotypes, which pose a difficult therapeutic challenge. In eosinophilic asthma co-occurring with allergic rhinitis, as well as in eosinophilic asthma associated with non-allergic rhinitis, eosinophils represent a key effector cell population driving the underlying type 2-mediated inflammatory response. The aim of this study is to analyze the efficacy of anti-IL5 or anti-ILR5 therapy in patients with severe eosinophilic asthma and persistent allergic or non-allergic rhinitis. **Methods:** In this single-center real-life study, the authors analyzed the effects of biological treatment on rhinological symptoms in patients over the age of 18 with severe uncontrolled eosinophilic bronchial asthma with coexisting persistent allergic or non-allergic rhinitis treated with mepolizumab or benralizumab. In all patients, the otolaryngologist performed anterior rhinoscopy. Evaluation of rhinological symptoms and quality of life in patients treated with anti-IL5 or anti-IL5 therapy before and six months after biological treatment was performed using the TNSS and SNOT-22 scales. **Results:** In total, 67 patients with eosinophilic severe bronchial asthma were included in the study; among them 39 (58.2%) suffered from persistent allergic rhinitis and 28 (41.8%) suffered from chronic non-allergic rhinitis. After six months of treatment, higher absolute differences for SNOT and TNSS were observed in the persistent allergic rhinitis group. **Conclusions:** Biological treatment with mepolizumab and benralizumab may reduce the severity of rhinological symptoms in both endotypes of inflammation. However, higher therapeutic benefits were observed in patients with co-existing persistent allergic rhinitis. It was demonstrated that, in addition to IgE-mediated responses, the eosinophil represented an important component of the inflammatory reaction in allergic rhinitis.

## 1. Introduction

Current medicine recognizes the necessity to determine the phenotype and endotype of the patient in order to personalize therapy, which allows for optimal therapeutic results to be obtained. Precision medicine means better disease control, more effective prevention, greater patient satisfaction, and socioeconomic cost savings [1]. Phenotyping, endotyping, and the search for biomarkers of the effectiveness of the therapy used also apply to the management of bronchial asthma, one of the most common chronic airway diseases [2].

The united airways concept suggests that patients with asthma typically exhibit parallel inflammation in the upper and lower airways [3]. According to this concept, inflammatory processes in the upper and lower airways are caused by a similar pathophysiological process, and the therapy used should be effective both for asthma and coexisting rhinological symptoms [4]. Patients with asthma have a higher incidence of allergic or non-allergic persistent rhinitis and chronic rhinosinusitis with or without nasal polyps than non-asthmatics [4]. Approximately half of patients with severe asthma and rhinological symptoms have an eosinophilic phenotype [5]. In eosinophilic asthma co-occurring with allergic rhinitis, as well as in eosinophilic asthma associated with non-allergic rhinitis, eosinophils constitute a central effector cell population orchestrating the type 2-driven inflammatory cascade. Their activity is sustained by IL-5-dependent survival and maturation signals, IL-4/IL-13-mediated amplification of airway hyperresponsiveness, and eotaxin-guided recruitment, collectively contributing to persistent airway inflammation. Therefore, phenotypes are characterized by a high number of eosinophils. Intensive research in recent years and understanding of crucial pro-inflammatory mediators have led to the development of biologic drugs specifically against them [6]. Because of the significant heterogeneity of this group of patients, many investigators focus their attention on the search for cellular markers of efficacy of biologic treatment [7,8]. Because the eosinophil is considered a key element in the inflammatory processes of the nasal mucosa currently, mepolizumab, an anti-IL-5 biologic or benralizumab anti-IL5R monoclonal antibody, may be a therapeutic option in this group of patients. The efficacy of these drugs for severe eosinophilic asthma as well as eosinophilic sinusitis with polyps has been confirmed in several clinical studies [9,10]. However, despite the identification of the eosinophilic phenotype and the use of targeted treatment, researchers also find that this therapy is not effective in some patients, which may support a mixed type of endotype inflammation due to the coexistence of other inflammatory factors in addition to the eosinophilic infiltrate in the lower and upper airways [11]. Therefore, there is a need for further research leading to an understanding of the inflammatory processes in the nasal mucosa that would allow for the effectiveness of the biological treatment to be predicted. Analysis of the outcomes of biological therapy in patients with eosinophilic/non-allergic and eosinophilic/allergic phenotypes may contribute to a better understanding of the pathophysiology of the inflammatory process and support the personalization of biologic treatment, enabling the achievement of optimal therapeutic effects.

The aim of this study is to analyze the efficacy of anti-IL5 or anti-ILR5 therapy in patients with severe eosinophilic asthma and persistent allergic or non-allergic rhinitis.

## 2. Materials and Methods

In this single-center real-life study, we analyzed the effects of biological anti-IL-5 or anti-IL5R treatment on rhinological symptoms and quality of life in patients over the age of 18 with severe uncontrolled eosinophilic bronchial asthma treated with mepolizumab or benralizumab with coexisting persistent allergic or non-allergic rhinitis. The data of patients treated with mepolizumab or benralizumab at the Department of Internal Medicine, Allergology and Clinical Immunology of the K. Gibinski University Clinical Hospital of the Silesian Medical University in Katowice, Poland were analyzed. Severe bronchial asthma and its eosinophilic phenotype were diagnosed based on the GINA criteria [12]. In accordance with current diagnostic criteria, chronic allergic rhinitis was diagnosed based on concordance between a documented history of symptom onset—including anterior or posterior rhinorrhea, nasal congestion or blockage, nasal pruritus, and sneezing—following allergen exposure and positive skin prick test results. Symptoms must occur more than 4 days per week and for more than 4 consecutive weeks per year. Non-allergic rhinitis was diagnosed in patients presenting with symptoms of rhinitis in the absence of clinical signs of infection and without evidence of systemic allergic sensitization, as indicated by negative skin prick test results to inhalant allergens [13]. The skin prick tests were performed according to the European Academy of Allergy and Clinical Immunology guidelines [14].

After an insightful differential diagnosis, the patients were then qualified for the therapeutic program according to the criteria of the National Health Fund (annex B.44). The patients were eligible for the treatment of severe eosinophilic asthma with anti-IL-5—mepolizumab—or anti-IL5R—benralizumab [15].

**Figure 1:**
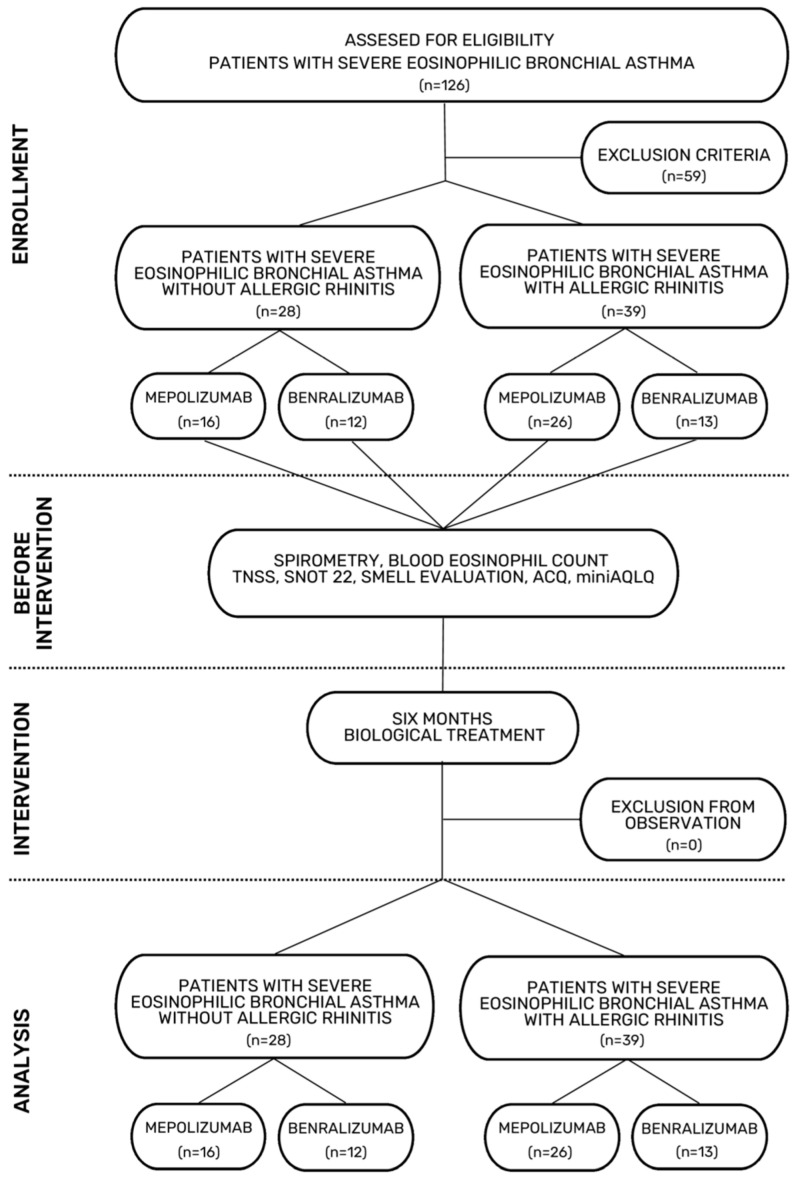
Flowchart of the study selection process.

The flowchart of the study selection process is shown in Figure 1.

### 2.1. Inclusion and Exclusion Criteria

The inclusion and exclusion criteria for biologic therapy for patients over the age of 18 with severe eosinophilic asthma according to the criteria of the National Health Fund (Annex B.44) are shown in Table 1.

In order to select the study group of patients with allergic or non-allergic rhinitis and severe eosinophilic asthma who qualified for biological treatment, supplementary allergological and otolaryngological parameters were used. The diagnosis of persistent allergic or non-allergic rhinitis was based on skin prick tests and clinical history (days with symptoms per week and number of consecutive weeks with symptoms according to ARIA) [16]. According to the EPOS 2020 guidelines, patients were allowed to use intranasal steroid in an equivalent dose to 200 µg of mometasone furoate nasal spray once daily during the study period [17]. Patients with rhinosinusitis exacerbation and the use of antileukotrienes or antihistaminic or systemic steroids less than 4 weeks prior to the initiation of the observation were excluded from the observation. Additionally, patients with recent endoscopic sinus surgery (within the past 6 months) and with clinically relevant nasal septum deviation were excluded from the observation.

### 2.2. Rhinological Evaluation

In all patients, the otolaryngologist performed anterior rhinoscopy.

### 2.3. Total Nasal Symptom Score (TNNS)

The Total Nasal Symptom Score (TNNS) was subjectively assessed based on the severity of symptoms such as nasal obstruction, secretion, sneezing, itching, and sleep disturbance. The severity of each symptom was based on the following scores: 0 = no symptoms; 1 = mild, unobtrusive symptoms; 2 = moderate, disturbing but tolerable symptoms; and 3 = severe, disturbing, perceived to interfere with daily activities/sleep and difficult-to-tolerate symptoms. The maximum Total Nasal Symptom Score is 15.

### 2.4. Sino-Nasal Outcome Test (SNOT-22)

The Sino-Nasal Outcome Test (SNOT-22) includes both classic nasal symptoms as well as extranasal symptoms such as poor sleep, ear/facial discomfort, and mood disturbance, features that have also been associated with persistent rhinological symptoms. The patients evaluated their symptoms over the preceding 2-week period. All questions are based on a 0–5 scale, where 0 defines no problems with the given symptom and 5 defines the maximum number of problems. The questionnaire results are included in the range of 0–110 points. Higher scores indicate a greater burden of symptoms and poorer quality of life.

### 2.5. Smell Assessment

Separately, the patients were asked about the presence of smell (1 = present; 0 = absent). The evaluation of the sense of smell was based on a question about the smell of coffee.

### 2.6. Eosinophil Count

Blood samples were collected in a hematologic sample tube containing anticoagulant, and eosinophil absolute counts was recorded using a Haematology Analyzer Sysmex XN-1000 (Sysmex Europe Corporation, Norderstedt, Germany).

### 2.7. Spirometry

A spirometry assessment was performed using the Ganshorn Medizin Electronic spirometer (SpiroScout, Niederlauer, Germany) according to the ATS/ERS technical guidelines [18]. FEV1 (forced expiratory volume in the first second), FVC (forced vital capacity), and the FEV1/FVC ratio were evaluated.

### 2.8. Asthma Control Questionnaire—Version 7 (ACQ-7)

This tool was used to assess asthma control in individuals over the previous week. It includes 7 items, covering symptoms, activity limitations, lung function, one question about rescue medication use, and one question about actual FEV1% predicted value. Each item is scored from 0 (no impairment) to 6 (maximum impairment). The total score is the average of all items. Interpretation: 0.75—well-controlled asthma, 0.75–1.5—not well controlled, and >1.5—poorly controlled.

### 2.9. Mini Asthma Quality of Life Questionnaire (miniAQLQ)

This questionnaire measures the impact of asthma on a patient’s quality of life over the past 2 weeks. It consists of 15 questions in four domains (symptoms—5 items, activity limitations—4 items, emotional function—3 items, and environmental stimuli—3 items). Patients rate items on a 7-point scale, where 1 = severely impaired and 7 = not impaired at all. Total score is the average of all items. A score change of 0.5 or more is considered a clinically meaningful improvement or decline. The higher the score in the MiniAQLQ, the better the quality of life of a patient.

TNNS, SNOT-22, smell evaluation, spirometry, blood eosinophil count, and the ACQ and miniAQLQ were assessed on two occasions: immediately before the first administration of the biological drug and after 6 months of therapy.

### 2.10. Statistical Analysis

The results are expressed as absolute numbers and percentages for frequencies, and mean ± standard deviations (if normally distributed) or median and interquartile range (if not normally distributed). Normality was assessed using the Shapiro–Wilk test. Quantitative variables were compared using the Wilcoxon signed-rank test or Mann–Whitney U-test. All analyses were performed with a software package (STATISTICA 14.0.1.25, StatSoft Poland). *p*-values of less than 0.05 were considered significant.

## 3. Results

**Figure 2:**
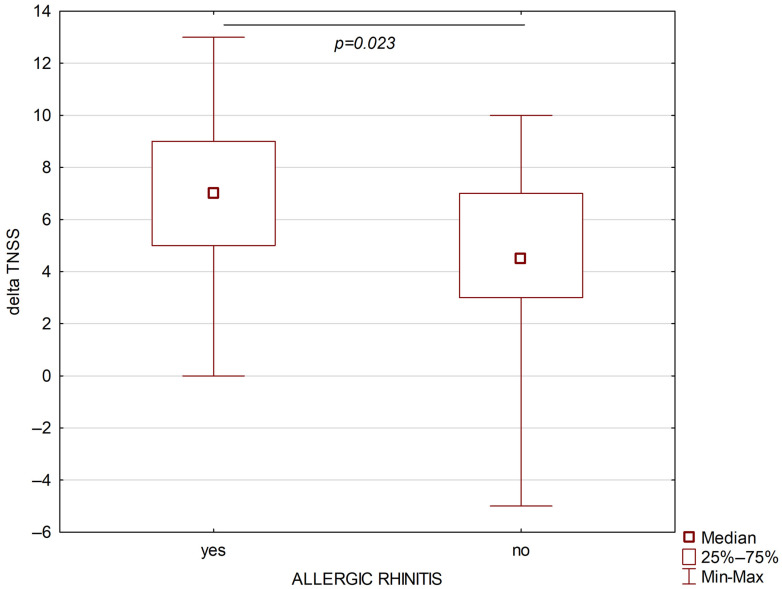
Changes in TNSS after six months of biological treatment of asthmatic patients with allergic rhinitis (left part) and non-allergic rhinitis (right part).

**Figure 3:**
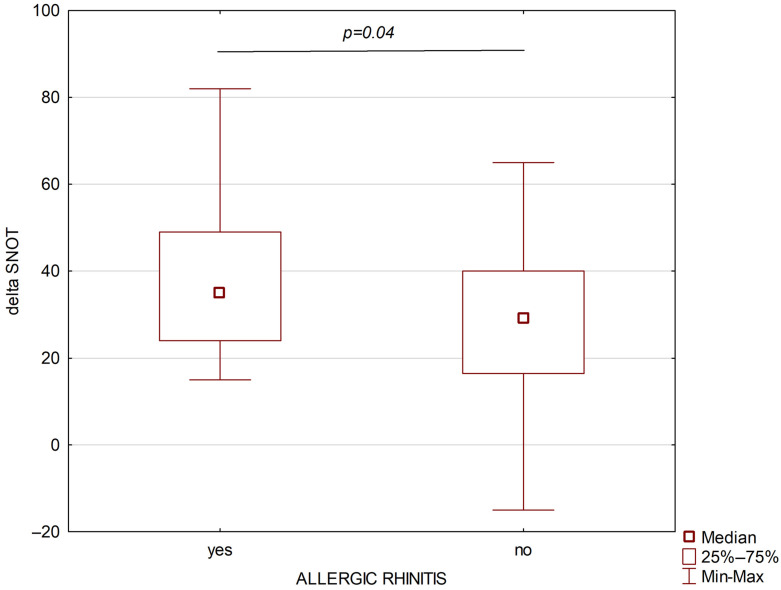
Changes in SNOT after six months of biological treatment of asthmatic patients with allergic rhinitis (left part) and non-allergic rhinitis (right part).

A total of 67 patients with severe bronchial asthma were included in the study; among them, 39 (58.2%) suffered from allergic rhinitis and 28 (41.8%) suffered from persistent non-allergic rhinitis. The study group included patients with monosensitization to house dust mites. The severity of allergic rhinitis in the studied patient cohort was classified as moderate to severe according to the ARIA guidelines [16]. Both groups were comparable in terms of age, sex, and BMI. The following parameters were assessed before the treatment: blood eosinophil absolute and percentage number, ACQ, AQLQ, SNOT, FEV1/FVC, TNSS values, FEV1, and FVC were higher in the allergic rhinitis group than in the non-allergic rhinitis group. After six months of treatment, higher absolute differences for SNOT and TNSS were observed in the persistent allergic rhinitis group (Figure 2 and Figure 3).

Both SNOT and TNSS decreased significantly after six months of treatment with both mepolizumab (for both parameters *p* < 0.00001) and benralizumab (*p* = 0.000016 and *p* = 0.000012, respectively).

Detailed data are shown in Table 2.

When the groups with persistent allergic or non-allergic rhinitis were further subdivided according to mode of biological treatment, the initial TNSS values were higher in the mepolizumab group both in the persistent allergic and non-allergic rhinitis subgroups. After treatment, the eosinophils values were lower in benralizumab-treated patients. The lowest changes in SNOT (delta SNOT) were observed in the benralizumab non-allergic rhinitis subgroup. However, no significant differences in TNSS or SNOT were found between drugs (presence or absence of eosinophilia in peripheral blood morphology) in the group with coexisting persistent allergic rhinitis (Table 3). Both the initial and after-treatment absolute number and percentages of blood eosinophils were comparable in the allergic rhinitis and non-allergic rhinitis groups (Table 2). The initial absolute number and percentages of blood eosinophils were also comparable after further subdivision of these groups according to the used biologic (mepolizumab vs. benralizumab); however, after 6 months of treatment, these parameters were significantly lower in patients treated with benralizumab, which is an expected treatment outcome (Table 3).

## 4. Discussion

The impaired drainage and ventilation within the nasal cavity and paranasal sinuses caused by heterogeneous inflammatory processes in the mucous membrane lead to chronic inflammation of the paranasal sinuses, which is a significant health, social, and economic problem [16,17]. Intranasal corticosteroid, short courses of oral corticosteroids, antihistamines, antibiotics, functional endoscopic sinus surgery, and immunotherapy are proposed for this heterogeneous group of patients [19,20]. Because of incomplete knowledge regarding the etiology and pathophysiology of rhinological symptoms, the treatment used to manage the disease in the international guidelines remains ineffective in some patients [17]. The most common inflammatory processes included in the group of T-2-dependent inflammations are chronic allergic rhinitis and eosinophilic rhinitis [5]. Immunotherapy, recommended for severe chronic allergic rhinitis, is a precise therapeutic method. In particular, molecular testing conducted prior to treatment qualification provides a high effectiveness. However, it is not effective for all patients [20]. Chronic eosinophilic inflammation is often associated with the occurrence of polyps in the paranasal sinuses and nasal cavities, which often requires surgical treatment [19]. Surgical treatment is not a causative treatment. The frequent reoperations necessary in this group of patients impair the physiological functions of the nasal cavity and paranasal sinuses and create a risk of orbital and intracranial complications [21]. Therefore, the inflammatory processes in the nasal mucosa and paranasal sinuses are a current research question. According to the concept of one way, one disease, the inflammatory process in bronchial asthma and patient’s rhinological symptoms are caused by the same endotype of the inflammatory process [22]. Due to the assumption of united airways, the effects of biological treatment in severe bronchial asthma may allow for us to gain an understanding of the inflammatory processes in the nasal mucosa. The effectiveness of biological treatment in the course of eosinophilic bronchial asthma and rhinological disorders has been confirmed in several studies [23,24]. However, a meta-analysis has also been published that indicates the limited effectiveness of this therapy. The non-response rate to biological treatment in the group of patients with eosinophilic chronic sinusitis with polyps was 48% for mepolizumab [11]. These data confirm the cellular heterogeneity of endotypes of rhinological diseases, which require further characterization in order to select the most effective biological drug for each patient. The effects of biological treatment may be particularly interesting in the case of mixed endotypes, which pose a difficult therapeutic challenge. Our analysis attempted to assess whether chronic allergic rhinitis coexisting with eosinophilic inflammation may affect the effects of anti-IL-5 (mepolizumab) or anti-IL5R (benralizumab) monoclonal antibody biological treatments. The recommended TNSS and SNOT-22 questionnaires were used to assess the severity of rhinological symptoms [25,26]. Before starting the biological treatment, a significantly higher severity of rhinological symptoms was found in the group of patients with coexisting chronic allergic rhinitis. After six months of biological therapy, a reduction in rhinological symptoms was observed in both the group with coexisting chronic allergic rhinitis and the group with non-allergic rhinitis. However, higher absolute differences for SNOT and TNSS were observed in the allergic rhinitis group. The results obtained suggest that during biological therapy with anti-IL-5 or anti-IL5R of the eosinophilic inflammatory process associated with chronic allergic rhinitis in the nasal mucosa, the role of eosinophils, which are also crucial in the late- phase reaction in chronic allergic rhinitis, is reduced. Our observation confirms the observations of other researchers regarding the role of eosinophilia in the late-phase reaction in allergic rhinitis [27,28]. The effectiveness of mepolizumab in treating eosinophilic asthma, both with and without an allergic component, has also been confirmed in a recent real-life study [29]. The authors noted that the group of patients with persistent allergic rhinitis treated with benralizumab, in whom no eosinophilia was found in peripheral blood morphology after biological treatment, did not differ in terms of the severity of rhinological symptoms from patients with persistent allergic rhinitis treated with mepolizumab, who were found to have eosinophilia after treatment.

## 5. Conclusions

Our study demonstrates the important role of eosinophils in both mixed endotypes: eosinophilic/allergic and non-allergic eosinophilic rhinitis. Treatment with the biologics mepolizumab or benralizumab may reduce the severity of rhinological symptoms in both inflammatory endotypes. Importantly, greater therapeutic benefits were observed in patients with concomitant chronic allergic rhinitis. The authors’ preliminary observation (no differences in the severity of rhinological symptoms in the group of patients with persistent allergic rhinitis and the presence or absence of eosinophilia after biological treatment) indicates the validity of analyzing mechanisms other than eosinophilic inflammation and allergy in the inflammatory process of the nasal mucosa. It also seems important to analyze additional factors such as viruses, bacteria, air pollutants, and other environmental irritants. Our observations indicate the need for further research into endotypes of chronic rhinitis in order to achieve optimal therapeutic options for patients.

## Figures and Tables

**Table 1:** Inclusion and exclusion criteria of biological treatment with mepolizumab or benralizumab based on the National Health Fund (Annex B.44).

Inclusion Criteria	Exclusion Criteria
1. Severe, uncontrolled bronchial asthma. 2. High doses of inhaled corticosteroids (>1000 mcg of beclomethasone dipropionate per day or another inhaled corticosteroid at an equivalent dose) in combination with another asthma control medication (long-acting β-2 adrenergic receptor agonist, leukotriene, theophylline derivative). 3. Two or more exacerbations in a year requiring systemic corticosteroids or an increase in their dose, which they use chronically. 4. Eosinophil count of ≥350 cell/µL during qualification or in the 12 months preceding qualification, or ≥150 cell/µL if systemic steroids are required for 6 months—5 mg daily prednisone, and the cumulative annual dose is ≥1.0 g. 5. Fulfilled at least 2 of the following criteria: a. Score in Asthma Control Questionnaire (ACQ) > 1.5 points; b. Hospitalization in the last 12 months due to an exacerbation of asthma; c. A life-threatening asthma attack incident; d. Persistent pulmonary obstruction (FEV1 < 80% from baseline or daily PEF variation > 30%); e. Impaired quality of life due to asthma (miniAQLQ < 5.0). 6. Non-smoker.	1. Contraindication to mepolizumab or benralizumab treatment. 2. Pregnancy. 3. Lactation period. 4. Parasitic infection. 5. Ongoing therapy with immunosuppressive or anti-cancer drugs, infusions of immunoglobulins or other biological treatments.

**Table 2:** Clinical and laboratory characteristic of patients with severe eosinophilic asthma with non-allergic or allergic rhinitis before treatment (0) and after 6 months (1). Data are presented as mean and standard deviations or median values and interquartile ranges or absolute and percentage. Bold values are significant.

	Allergic Rhinitis N = 39	Non-Allergic Rhinitis N = 28	*p*
Age, years	54.7 ± 12.8	58.9 ± 13.8	0.23
Female, n (%)	23 (82)	29 (76)	0.55
BMI-0	27.2 ± 5.7	27.9 ± 5.8	0.73
Eosinophils %-0	5.13 (3.4–8.2)	6.6 (4.3–9.65)	0.24
Eosinophils 1^3/uL0	0.38 (0.25–0.64)	0.515 (0.29–0.835)	0.11
FVC-0	80 (65–88)	64.5 (56.5–80.5)	**0.04**
FEV1/FVC-0	67 (56–72)	63.85 (52–72)	0.38
FEV1-0	65 (49–78)	52 (41.5–69)	**0.049**
ACQ-0	3.4 (3–4.6)	3.7 (3.35–4.1)	0.52
AQLQv	2.8 (2–3.3)	2.55 (2.05–3)	0.62
TNSS-0	13 (9–13)	9.5 (6–12)	**0.01**
SNOT-0	68 (55–82)	63.5 (50–81)	0.42
Eosinophils %-1	0.6 (0–1.4)	0.35 (0–1.1)	0.36
Eosinophils 1^3/uL-1	0.04 (0–0.80	0.025 (0–0.09)	0.61
FVC-1	83 (70–91)	80.5 (61–91.5)	0.48
FEV1/FVC-1	71 (60.1–77)	68(57.5–71.5)	0.2
FEV1-1	76 (56–84)	65.5 (44–80)	0.18
ACQ-1	2.4 (1.4–3.0)	1.8 (1.35–2.65)	0.23
AQLQ-1	4.1 (3.3–5.1)	4.4 (3.8–5.5)	0.28
TNSS1	5 (3–7)	4.5 (2.5–5)	0.46
SNOT1	30 (21–38)	32.5 (22.5–45)	0.23
Delta TNSS	7 (5–9)	4.5 (3–7)	**0.023**
Delta SNOT	35 (24–49)	29 (16.5–40)	**0.04**

**Table 3:** Clinical and laboratory characteristic of patients with severe eosinophilic asthma with non-allergic or allergic rhinitis divided according to mode of biological treatment before treatment (0) and after 6 months (1). Data are presented as mean and standard deviations or median values and interquartile ranges or absolute and percentage. Bold values are significant.

	Allergic Rhinitis Group			Non-Allergic Rhinitis Group		
Treatment	Mepolizumab 26 (62%)	Benralizumab 13 (52%)	*p*	Mepolizumab 16 (38%)	Benralizumab 12 (48%)	*p*
Parameters						
Age, years	53.5 ± 13	57 ± 12.2	0.49	54.8 ± 13	64.2 ± 13.5	0.07
BMI-0	26 ± 5.2	28.7 ± 6.5	0.32	28.6 ± 5.7	27 ± 6.2	0.7
Eosinophils %-0	6.2 (3.4–9.1)	4.4 (3.5–6)	0.15	7.3 (0.4–15.4)	5.9 (3.65–7.85)	0.17
Eosinophils 1^3/uL-0	0.4 (0.26–0.66)	0.38 (0.25–0.46)	0.57	0.54 (0.05–1.61)	0.49 (0.245–0.73)	0.4
FVC-0	81 (0.65–0.85)	76 (66–88)	0.1	60.5 (37–100)	67.5 (62–75)	0.68
FEV1/FVC-0	63 (56–72)	70 (64–71)	0.56	62.3 (52–68.5)	64.5 (52–75)	0.63
FEV1-0	63.5 (48–73)	75 (50–79)	0.58	52 (26–85)	55.5 (44–69)	0.66
ACQ-0	3.75 (3–4.6)	3.3 (2.7–3.4)	0.09	3.7 (2–5.7)	3.75 (3.25–4.05)	0.75
AQLQ-0	2.65 (2–3.1)	3 (2.4–3.4)	0.2	2.65 (1.2–4.1)	2.55 (2.25–3)	0.93
TNSS-0	13 (12–14)	10 (7–12)	**0.02**	12 (2–15)	7.5 (4.5–10)	**0.016**
SNOT-0	67 (58–85)	71 (55–73)	0.74	70 (31–98)	58 (46.5–66.5)	0.22
Eosinophils %-1	1.05 (0.6–1.8)	0 (0–0)	**0.00001**	1.05 (0.2–6)	0 (0–0)	**0.000004**
Eosinophils 1^3/uL-1	0.07 (0.04–0.1)	0 (0–0)	**0.00001**	0.07 (0.01–0.7)	0 (0–0)	**0.000004**
FVC1-1	83 (70–94)	82 (65–87)	0.65	74 (45–108)	83.5 (70–88.5)	0.61
FEV1/FVC-1	65.5 (59–77)	73 (70–77)	0.48	67 (65–71)	68.5 (49–72)	0.8
FEV1-1	69.5 (56–83)	77 (63–86)	0.27	60.5 (24–96)	73 (45–76.5)	0.88
ACQ-1	2.3 (1.5–3.7)	2.4 (1.4–2.7)	0.73	2 (0.1–4.1)	1.65 (1.4–2.3)	0.43
AQLQ-1	4.165 (3.4–5.4)	3.9 (3.3–4.4)	0.47	4.4 (2–6.7)	4.35 (3.95–5.5)	1.0
TNSS1	5 (4–7)	5 (3–5)	0.13	5 (0–10)	3.5 (0.5–5)	0.09
SNOT1	29.5 (21–36)	32 (23–38)	0.51	32.5 (5–69)	33.5 (29.5–42)	0.66
Delta TNSS	7 (5–9)	5 (4–7)	0.28	5.5 (−5–10)	4 (3–5)	0.11
Delta SNOT	41 (22–54)	33 (27–42)	0.45	33.5 (−15–65)	18.5 (12.5–29.5)	**0.04**

## Data Availability

The raw data supporting the conclusions of this article will be made available by the authors on request.

## Abbreviations

The following abbreviations are used in this manuscript:

FEV1	Forced Expiratory Volume
ACQ	Asthma Control Questionnaire
AQLQ	Asthma Quality of Life Questionnaire
OCS	Oral Corticosteroids
TNSS	Total Nasal Symptom Score
SNOT	Sino-Nasal Outcome Score
